# Context-aware digital nutrition and abdominal acoustic feedback in Taiwanese university students: a 12-week single-arm field intervention and a separate 4-week randomized feasibility pilot

**DOI:** 10.3389/fnut.2026.1853482

**Published:** 2026-09-07

**Authors:** Chuanchia Wang, Chen Han, Jwoshiun Sun, Tungjing Fang

**Affiliations:** 1 Department of Electronic Engineering, National Taipei University of Technology, Taipei, Taiwan; 2 Doctoral Program in Design, College of Design, National Taipei University of Technology, Taipei, Taiwan; 3 Department of Physiology and Biophysics, Graduate Institute of Physiology, College of Medicine, National Defense Medical University, Taipei, Taiwan

**Keywords:** abdominal acoustic sensing, digital nutrition, dietary self-regulation, eating behavior, human-computer interaction, mobile health, wearable sensors, university students

## Abstract

**Background:**

Most digital nutrition tools rely on retrospective meal logging and generic reminders. AbSound adds abdominal acoustic cues intended to support meal-timing reflection and dietary self-regulation. These cues were treated as meal-relevant behavioral proxies, not physiological markers of hunger, satiety, or gastrointestinal motility. We evaluated the platform among Taiwanese university students.

**Materials and methods:**

Phase 1 was a 12-week single-arm field intervention with 200 participants completing follow-up. Outcomes included engagement, meal-logging completeness, fruit and vegetable intake, dietary self-regulation, daily steps, WHOQOL-BREF, and BMI. Phase 2 was a separate 4-week open-label randomized feasibility pilot (*N* = 32; 16 per group) comparing AbSound with standard tracking. Analyses emphasized within-participant trajectories, feasibility estimates, exploratory between-group changes, and agreement with composite meal-state/acoustic-event labels.

**Results:**

In Phase 1, fruit and vegetable intake, dietary self-regulation, daily steps, and quality of life improved over time, whereas BMI remained stable. Because Phase 1 had no control group, these findings represent temporal associations. Active-day engagement was 78%, meal-logging completeness was 48.5%, and both declined across the semester. In Phase 2, participants used AbSound for 9.35 hours/day across 25.81 of 28 days and acknowledged 78.54% of alerts. Exploratory between-group change estimates directionally favored AbSound for late-night snacking, eating speed, diet quality, and eating self-regulation, but the pilot was not powered for efficacy. The acoustic model achieved 87.2% accuracy and a macro-F1 of 82.8% across 1,207 labeled events; these metrics reflect agreement with composite event labels rather than construct validation of hunger, satiety, or gut motility.

**Conclusions:**

The findings support the short-term feasibility of context-aware digital nutrition and abdominal acoustic feedback as behavioral self-regulation supports. Larger randomized trials should evaluate efficacy, time-lock acoustic events to validated appetite ratings, use participant-disjoint acoustic validation, and assess longer-term behavioral and clinical outcomes.

## 1 Introduction

Digital food environments combine food marketing, algorithmic ranking, mobile ordering, and 24-hour convenience. These exposures do not determine individual dietary behavior, but they can make palatable, low-effort options especially salient when time pressure, fatigue, or stress reduces self-regulatory capacity (1–5). Precision nutrition and artificial intelligence (AI) may improve the relevance of dietary guidance, yet field evidence remains limited on whether context-aware, body-proximal feedback can support eating-related decisions without becoming intrusive or implying unsupported physiological inference (6–8).

Consumer wearables and dietary self-monitoring systems can increase awareness, reduce recall burden, and provide feedback, but their impact is constrained by declining engagement, reporting burden, and the fact that digital tools generally facilitate rather than independently drive behavior change (9–13). The design challenge is therefore not merely to collect more dietary data, but to deliver interpretable feedback at an opportune moment, in a tolerable form, with enough friction to support reflection without creating excessive burden.

The present study considers university students as a relevant population for examining this problem. The rationale for selecting this demographic is 3-fold: first, dietary behavior during young adulthood is shaped by time scarcity, stress, living arrangements, meal skipping, financial constraints, and increasing autonomy over food choice (14–16). Taiwan-specific studies have demonstrated that the adoption of a healthy diet among university students is associated with nutrition literacy, eHealth literacy, self-schema, family and food-environment factors, cooking frequency, and expenditure patterns (17–19). Consequently, Taiwanese university students provide a culturally specific test setting characterized by dense convenience stores, night markets, food delivery, and late-night academic routines. It is recommended that generalization be limited to comparable young-adult, digitally literate populations.

The physiological rationale for abdominal acoustic feedback is grounded in gut-brain appetite biology, though it is not equivalent to it. Meal initiation, continuation, and termination are governed by gastrointestinal signals, vagal pathways, endocrine cues, reward processing, interoception, habit, and cognitive control (20–25). Interventions that direct users' attention to internal hunger, fullness, and satisfaction cues can support adaptive eating, provided that prompts are non-punitive and non-surveillance-oriented (26–29). In the present study, abdominal acoustic events are operationally defined as meal-relevant acoustic proxies. They are not continuous gut-motility measures and have not been validated as biomarkers of hunger.

To avoid theoretical overloading, we employ the HCI framework as a layered explanation rather than a set of competing models. Technology acceptance constructs address adoption and trust. Effective engagement and just-in-time adaptive intervention concepts address timing and context. Kano theory addresses how feature expectations may shift after repeated use, and behavior change theory addresses self-efficacy, feedback, habit, and control processes (30–43). Together, these layers establish a correlational account linking interface adoption, cue timing, and self-regulation. They do not imply that user acceptance alone proves behavioral efficacy.

Within this framework, AbSound is positioned as a behavioral support layer. Meal-relevant acoustic events may help align feedback with eating episodes, post-meal reflection, and pace awareness. The proposed mechanism concerns cue interpretation and self-regulatory reflection, not diagnosis, appetite-hormone or microbiome inference, or direct measurement of gastrointestinal motility.

Research conducted to date on abdominal sounds has yielded progress in the following areas: automated bowel-sound detection; the design of contact-acoustic sensors; analysis using smartphones; multichannel wearable abdominal sensors; and machine-learning recognition under conditions of noise and class imbalance (44–50). The primary focus of these systems has been on bowel-sound detection, monitoring digestive system activity, clinical or preclinical monitoring, and identifying events using algorithms. AbSound is distinguished by its intended application; it incorporates a contact-acoustic cue stream within a digital nutrition/HCI workflow to facilitate eating-related timing and reflection. The present data, therefore, demonstrate the novelty of the method as translational and interactional rather than diagnostic; the feasibility and meal-state/acoustic-event agreement of the method are evaluated, rather than the validation of a physiological measurement instrument.

The study used a two-phase evidence sequence. Phase 1 estimated 12-week engagement and within-participant changes in dietary self-regulation, fruit and vegetable intake, activity, quality of life, and BMI in a single-arm field intervention. Phase 2 estimated feasibility, event-level label agreement, and short-term directional behavioral signals relative to standard tracking in a randomized pilot. Neither phase tested a gut-brain mechanism or clinical efficacy, and Phase 1 was not designed for causal attribution

The evidence sequence is divided into two phases, and the inferential boundary is defined accordingly. Phase 1 field intervention: objective = estimate 12-week engagement, logging, dietary self-regulation, fruit/vegetable intake, activity, quality of life, and BMI change; hypothesis status = descriptive/pragmatic, not controlled efficacy; primary outcomes = within-participant change and weekly/biweekly process trajectories; inferential boundary = no causal attribution because no control group was used. The CEHSV-KANO-TAM-SEM adoption framework provides an associative model-based interpretation of adoption, perceived usefulness, ease of use, feature valuation, and contextual pathways. AbSound validation: objective = assess device feasibility and event-level agreement for meal-relevant acoustic labels; hypothesis status = preliminary; inferential boundary = no participant-disjoint external AI validation and no direct physiological validation of hunger, satiety, gastric emptying, or gut motility. Phase 2 randomized pilot: objective = estimate feasibility and directional behavioral signal over 4 weeks; inferential boundary = not definitive clinical, metabolic, or obesity-prevention efficacy. Table 1 summarizes the evidence architecture and inferential boundaries for the Phase 1 field intervention, the adoption model, AbSound event agreement, and the Phase 2 randomized feasibility pilot.

**Table 1:** Evidence architecture and inferential boundaries.

Evidence component	Primary question	Primary estimands/metrics	What the design supports	What it cannot support
Phase 1 single-arm field intervention	How do engagement and self-regulation indicators change over 12 weeks?	Within-participant change: weekly/biweekly mixed-effects trajectories	Feasibility, retention, process trajectories, and descriptive longitudinal associations	Controlled efficacy or attribution to any single intervention component
CEHSV-KANO-TAM-SEM adoption framework	How do perceived usefulness, ease of use, context, and feature valuation relate to use and outcomes?	SEM paths, subgroup invariance, baseline-to-week-12 Kano reclassification	Associative HCI interpretation of adoption and feature expectations	Causal mediation, proof of mechanism, or proof of durable behavior change
AbSound event validation	Can the acoustic workflow classify meal-state/acoustic-event labels?	Accuracy, balanced accuracy, macro-F1, class-specific precision/recall/specificity/F1	Preliminary analytical agreement with composite reference labels	Participant-disjoint external AI validation or physiological validation of hunger, satiety, gastric emptying, or gut motility
Phase 2 randomized feasibility pilot	Is AbSound feasible, and does it show a directional behavioral signal over 4 weeks?	Wear time, days worn, alert acknowledgment, change scores, ANCOVA sensitivity estimates.	Feasibility and planning estimates for a future RCT	Definitive clinical, metabolic, weight-loss, or obesity-prevention efficacy

## 2 Materials and methods

The manuscript analyzes two empirical phases of the same human-centered digital health program. Phase 1 was a 12-week prospective single-arm field intervention using a wearable-supported mobile dietary platform among Taiwanese university students. Phase 2 was a separate 4-week open-label randomized feasibility pilot comparing AbSound with standard tracking. The phases address distinct questions: longitudinal field implementation in Phase 1 and pilot feasibility plus directional behavioral signals in Phase 2.

The Phase 1 cohort and the CEHSV-KANO-TAM-SEM adoption framework have been described in prior technology-adoption-focused analyses. In the present manuscript, reused elements are limited to the Phase 1 cohort description, pre-specified CEHSV constructs, global SEM path estimates, and baseline/week-12 Kano endpoint classifications (51). The new contribution is the integration of baseline-to-week-12 inferential statistics with effect sizes and confidence intervals, weekly and biweekly longitudinal process analyses, Phase 2 randomized pilot change-score and ANCOVA analyses, event-level AbSound validation metrics, and a nutrition/HCI interpretation focused on behavioral self-regulation rather than technology adoption alone.

Phase 1 of the study was conducted at a major university in northern Taiwan. Participants were recruited in a university setting, and screening was conducted before enrolment. Following screening of 248 students, 212 met the eligibility criteria and completed the baseline assessment. Furthermore, 200 participants completed the 12-week follow-up assessment, resulting in an overall retention rate of 93% among those who had undergone the baseline assessment. The study's participation criteria included university students aged 18 to 30 who could provide informed consent and use the mobile/wearable study platform. The participants in this study ranged in age from 18 to 30 years (mean age: 20.4 ± 1.8 years), with 62% of the sample identifying as female. The participants' residential status was as follows: 107 resided in dormitories, while 93 lived with family. The study protocol was approved by the university institutional review board (IRB No. KY2021-609), and all participants provided informed consent.

Phase 2 comprised 32 participants assigned 1:1 to AbSound or standard tracking after baseline assessment. The allocation sequence was generated before intervention assignment using a computer-based random-number procedure with equal allocation, and assignment was implemented after completion of baseline measures. Participants and field staff could not be blinded after allocation because AbSound was visible and required active wear. The analytic dataset retained all randomized participants (16 per group), with no post-randomization exclusions. Allocation concealment beyond baseline, before disclosure, was not independently audited; therefore, the pilot is reported as an open-label randomized feasibility study rather than a masked efficacy trial.

Reference labels for AbSound validation were generated through a composite annotation workflow. Candidate windows were identified from timestamped meal logs, participant-reported meal starts or eating episodes, prompt-delivery records, sensor recordings, and artifact flags. Trained annotators reviewed each acoustic segment against its time-frequency trace and contextual timestamps and assigned one of four study-defined categories: meal_start, eating_episode, post_meal_fullness, or non_eating_gut_sound. Segments with substantial motion artifact, ambiguous context, or inadequate signal-to-noise ratio were flagged before metric calculation. The label post_meal_fullness denotes a post-meal contextual category, not a subjective fullness rating. No time-locked validated hunger or satiety ratings were available; therefore, construct validity for appetite states could not be tested.

The Phase 1 platform incorporated a combination of features, including wearable-supported activity capture, a mobile dietary interface, low-friction meal logging, personalized goals, nutrition-balance feedback, contextual prompts, and optional social or reward features. The feature prioritization process employed Kano logic, in which reliability, synchronization, and battery visibility were designated as fundamental requirements. Meal logging, personalized goals, and nutrition-balance feedback were categorized as performance functions, while rewards, social sharing, recipe sharing, and voice guidance were identified as potential attractive features.

The Phase 2 AbSound workflow added a body-proximal acoustic-sensing layer over the epigastric/upper abdominal region. The event-level validation export included reference labels, model predictions, prediction probabilities, alert delivery, alert type, acknowledgment, sensor location, root-mean-square (RMS) amplitude, spectral centroid, low-frequency band power (20–200 Hz), signal-to-noise ratio, and artifact flags. Algorithmic performance is therefore defined as agreement with meal-relevant event labels. The acoustic classifier is not described as a diagnostic model, direct hunger detector, or independently validated gut-motility instrument. AbSound is a skin-coupled contact-acoustic platform comprising an acoustic coupling interface, a stethoscope-like or silicone/gel-assisted contact layer, an AbMT-like contact-transducer sensing module, signal-conditioning electronics, digitization, local storage or wireless transfer, and app/HMI interaction.

Because alert timing was based on acoustic/contextual labels rather than validated appetite states, prompts were framed as optional reflective cues. Hunger-timing prompts were linked to meal-start and eating-episode contexts, whereas fullness/pace prompts were linked to post-meal or rapid-eating contexts. The app invited participants to pause, assess their internal state, eat more slowly, or consider an alternative snack; it did not assert that they were hungry or full or prescribe caloric restriction.

Phase 1 outcomes included active days per week, meal-logging completeness, daily fruit and vegetable servings, dietary self-regulation on a 0-100 scale, daily steps, WHOQOL-BREF overall score, and BMI. The scoring of the WHOQOL-BREF followed the established framework for quality-of-life instruments (51). The analysis of meal-logging completeness was conducted as an implementation outcome, rather than as a missing completely at random phenomenon.

Phase 2 participant-level outcomes included late-night snack episodes per week, eating speed (1–5), diet quality (0–100), self-regulation of eating behavior (SREB), weight, and waist circumference. Device metrics included daily wear time, days worn, hunger-timing and fullness/pace alerts, acknowledgment rate, detected eating episodes, and participant-level algorithmic accuracy. Event-level validation used the four study-defined meal-state/acoustic-event labels described above.

AbSound used 44.1-kHz, 24-bit recordings with 25-ms frames, a 10-ms hop length, normalization, pre-emphasis, windowing, and short-time Fourier transform (STFT) representations. These settings define the implemented pilot specification and remain candidates for future ablation because sampling rate, filtering, and window length affect battery demand, storage, artifact sensitivity, and event boundaries.

Feature extraction and export included RMS amplitude, spectral centroid, 20–200-Hz band power, signal-to-noise ratio, prediction probabilities, sensor location, alert delivery, alert type, acknowledgment, and artifact flags. Segments were assigned the study-defined labels meal_start, eating_episode, post_meal_fullness, or non_eating_gut_sound; these labels represent meal-related acoustic contexts rather than validated appetite, gastric-emptying, or motility measures.

The primary analysis of the baseline-to-week-12 Phase 1 comparisons used paired *t*-tests. The normality of change scores was assessed using histograms, Q-Q plots, and Shapiro-Wilk tests. Given that *n* = 200, paired *t*-tests were retained as the primary estimator of mean change, and Wilcoxon signed-rank tests were added as non-parametric sensitivity analyses. The mean changes, 95% confidence intervals, *p*-values, and Cohen's dz effect sizes are reported.

Weekly trajectories were analyzed using random-intercept linear mixed-effects models with fixed effects for study week and the mid-semester window and a participant-level random intercept. Weeks 6–8 defined the mid-semester period. Biweekly summaries were calculated as participant-level means within two-week blocks and then aggregated across participants. SEM and subgroup-invariance results were interpreted using conventional fit-index and invariance-testing criteria (52).

Meal-logging completeness was treated as an implementation exposure and a potential source of adherence bias. A formal post hoc adherence-stratified or inverse-probability weighted outcome model was not performed because the available revision dataset did not retain participant-level meal-entry exposure linked to every outcome record with sufficient granularity to estimate adherence-adjusted effects without imposing post-treatment selection assumptions. The manuscript therefore reports logging completeness transparently, interprets Phase 1 estimates as susceptible to adherence bias, and specifies adherence-stratified, per-protocol, and exposure-response analyses as requirements for the next adequately powered randomized trial.

The inferential hierarchy was specified as follows: Phase 1 baseline-to-week-12 paired estimates and weekly mixed-effects slopes are descriptive longitudinal estimands; Wilcoxon tests and ANCOVA models are sensitivity analyses; SEM and subgroup contrasts are exploratory explanatory analyses; Kano reclassification is descriptive feature-valuation evidence; logging completeness is an implementation exposure and potential bias mechanism; AbSound event metrics are preliminary analytical-validation estimates; and Phase 2 between-group change estimates are pilot feasibility and directional behavioral estimates. No Phase 1 estimate is interpreted as controlled efficacy, and no Phase 2 estimate is interpreted as definitive clinical or metabolic efficacy.

For Phase 2, baseline comparability was described using Welch's *t* tests. Primary pilot comparisons used baseline-to-follow-up change scores, Welch's *t* tests, and 95% confidence intervals; ANCOVA models with follow-up outcome as the dependent variable, baseline outcome as a covariate, and group as the predictor served as sensitivity analyses. *P* values and Hedges g are reported for estimation and trial planning, not confirmatory efficacy testing, and no multiplicity-adjusted conclusion is drawn (54–57).

The Phase 2 sample size was set by feasibility and resource constraints rather than a confirmatory efficacy calculation. A two-sided sensitivity power analysis under an equal-allocation independent-samples *t*-test model (α = 0.05; *n* = 16 per group) indicated 80% power only for a standardized mean difference of Cohen's *d* = 1.02; approximate power was 27.8% for *d* = 0.50 and 59.1% for *d* = 0.80. The pilot was therefore underpowered for small-to-moderate between-group effects, and all group comparisons were treated as exploratory directional estimates.

AbSound event validation was summarized using overall accuracy, balanced accuracy, macro-F1, and class-specific precision, recall, specificity, and F1. Because events were clustered within participants and reference labels were study-defined meal-state/acoustic-event annotations rather than independent physiological ground truth, these estimates are descriptive. Participant-level accuracy is reported as mean ± SD. Self-report and dietary-assessment limitations were interpreted in light of established nutrition-assessment concerns (57–59).

## 3 Results

### 3.1 Participant flow, adherence, and semester-long engagement

**Figure 1:**
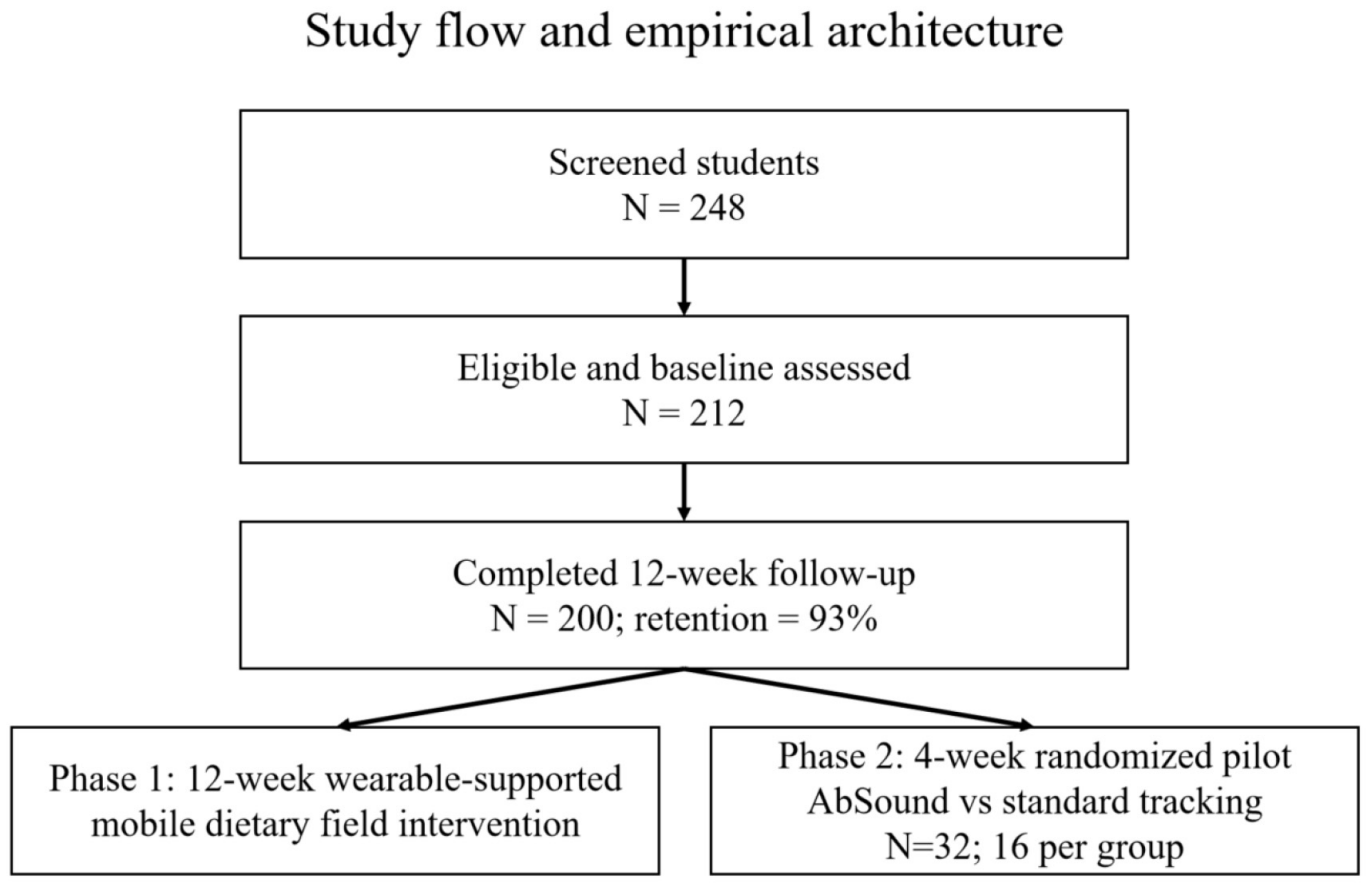
Study flow and two-phase empirical architecture. Phase 1 was a 12-week field intervention; Phase 2 was a separate 4-week randomized feasibility pilot of AbSound.

As illustrated in Figure 1, the 12-week field intervention can be considered separately from the 4-week AbSound randomized feasibility pilot.

### 3.2 Phase 1 showed within-participant improvements in fruit and vegetable consumption, dietary self-regulation, daily steps, and WHOQOL-BREF

Phase 1 retention was high, with 200 of 212 participants who were assessed at baseline completing the week-12 assessment. The overall active-day engagement rate was 78%, the overall meal-logging completeness rate was 48.5%, and the System Usability Scale score was 71.52. The 48.5% meal-logging completeness value is now interpreted as both a useful exposure metric and a potential source of adherence bias, rather than a negligible implementation issue.

The results presented in Table 2 support feasibility and retention in a Taiwanese university student context. They do not establish controlled efficacy because Phase 1 had no parallel control group.

**Table 2:** Phase 1 participant profile and implementation indicators.

Indicator	Value
Screened students	248
Eligible and baseline assessed	212
Completed 12-week follow-up	200
Retention	93%
Age, years	20.4 ± 1.8
Female	62%
Dormitory residence	107 (53.5%)
Home/family cohabitation	93 (46.5%)
Active-day engagement	78% of study days
Meal-logging completeness	48.5%
System usability scale	71.52
BMI at baseline and follow-up	22.41 ± 3.09 kg/m^2^

The weekly process data showed two concurrent descriptive patterns: engagement declined gradually, while mean dietary, activity, and quality-of-life indicators improved. The mean number of active days decreased from 6.00 (95% CI 5.90–6.10) in week 1 to 4.75 (95% CI 4.64–4.85) in week 12. Meal-logging completeness decreased from 57.0% to 45.5%, with the largest decrease around mid-semester. Fruit and vegetable intake increased from 3.22 to 4.31 servings per day, dietary self-regulation from 64.41 to 78.97, steps from 6,235 to 8,293 steps per day, and WHOQOL-BREF from 70.84 to 77.32. These patterns should be read as single-arm process trajectories rather than evidence that the platform alone caused the changes.

**Figure 2:**
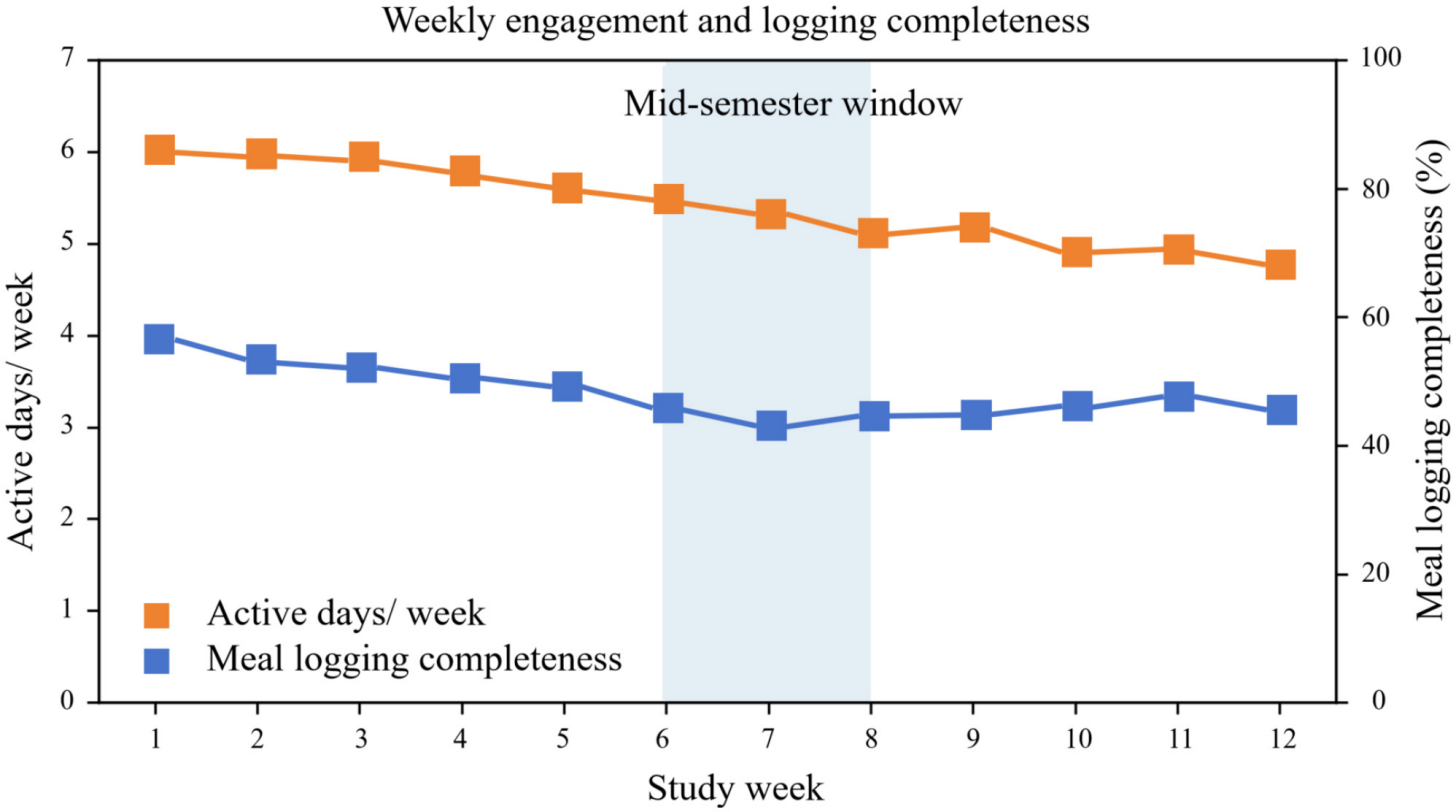
Weekly active-day engagement and meal-logging completeness with 95% confidence intervals. The shaded area marks the mid-semester window.

As illustrated in Figure 2, engagement and logging persisted across the semester but declined gradually, with a mid-semester attenuation in meal logging. The findings do not identify a specific academic factor, device feature, or intervention component as the cause of the decline.

**Figure 3:**
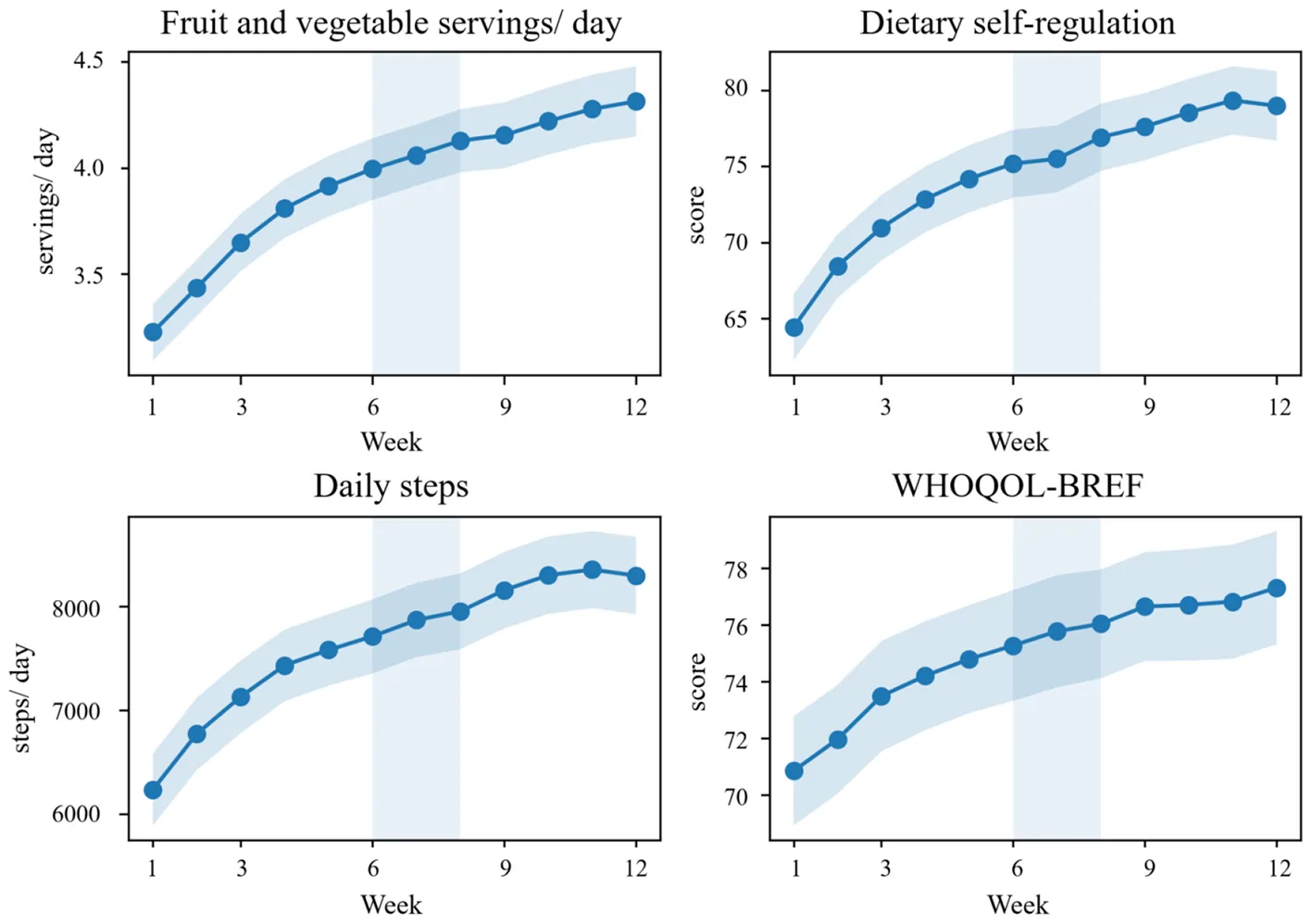
Weekly behavioral and quality-of-life trajectories with 95% confidence intervals.

Figure 3 shows progressive changes in behavioral and quality-of-life indicators with uncertainty intervals. These single-arm trajectories do not establish clinical efficacy or identify an active component of the multicomponent Phase 1 platform.

Table 3 summarizes the weekly patterns quantified in the mixed-effects models; individual trajectories may differ from the group mean.

**Table 3:** Weekly phase 1 longitudinal outcomes. Values are means with 95% confidence intervals.

Week	Active days M (95% CI)	Meal logging M (95% CI)	Fruit/veg M (95% CI)	Self-regulation M (95% CI)	Steps M (95% CI)	WHOQOL M (95% CI)
1	6.00 (5.90, 6.10)	0.570 (0.553, 0.586)	3.22 (3.09, 3.36)	64.41 (62.23, 66.59)	6,235 (5,889, 6,580)	70.84 (68.91, 72.77)
2	5.94 (5.84, 6.04)	0.535 (0.517, 0.553)	3.43 (3.30, 3.57)	68.44 (66.35, 70.53)	6,772 (6,425, 7,119)	71.96 (70.03, 73.88)
3	5.91 (5.81, 6.01)	0.517 (0.501, 0.534)	3.65 (3.51, 3.78)	70.96 (68.81, 73.11)	7,128 (6,778, 7,478)	73.49 (71.54, 75.43)
4	5.76 (5.66, 5.85)	0.502 (0.485, 0.519)	3.81 (3.67, 3.95)	72.83 (70.68, 74.99)	7,431 (7,086, 7,775)	74.20 (72.28, 76.13)
5	5.59 (5.48, 5.70)	0.490 (0.472, 0.508)	3.91 (3.77, 4.06)	74.18 (71.97, 76.39)	7,578 (7,234, 7,921)	74.79 (72.89, 76.70)
6	5.47 (5.36, 5.58)	0.456 (0.438, 0.473)	3.99 (3.85, 4.14)	75.17 (72.94, 77.40)	7,709 (7,352, 8,065)	75.27 (73.32, 77.23)
7	5.32 (5.21, 5.42)	0.428 (0.410, 0.447)	4.06 (3.92, 4.20)	75.49 (73.29, 77.69)	7,866 (7,508, 8,224)	75.78 (73.79, 77.76)
8	5.10 (4.98, 5.22)	0.443 (0.427, 0.460)	4.13 (3.98, 4.28)	76.91 (74.70, 79.12)	7,950 (7,586, 8,313)	76.04 (74.12, 77.96)
9	5.17 (5.05, 5.28)	0.448 (0.430, 0.466)	4.15 (4.00, 4.31)	77.60 (75.40, 79.80)	8,153 (7,784, 8,521)	76.65 (74.73, 78.57)
10	4.89 (4.79, 4.99)	0.458 (0.441, 0.475)	4.22 (4.06, 4.38)	78.53 (76.31, 80.75)	8,297 (7,924, 8,670)	76.71 (74.74, 78.68)
11	4.93 (4.82, 5.04)	0.475 (0.457, 0.492)	4.28 (4.12, 4.44)	79.33 (77.09, 81.57)	8,351 (7,981, 8,721)	76.82 (74.81, 78.84)
12	4.75 (4.64, 4.85)	0.455 (0.437, 0.472)	4.31 (4.15, 4.48)	78.97 (76.67, 81.26)	8,293 (7,922, 8,664)	77.32 (75.32, 79.31)

**Figure 4:**
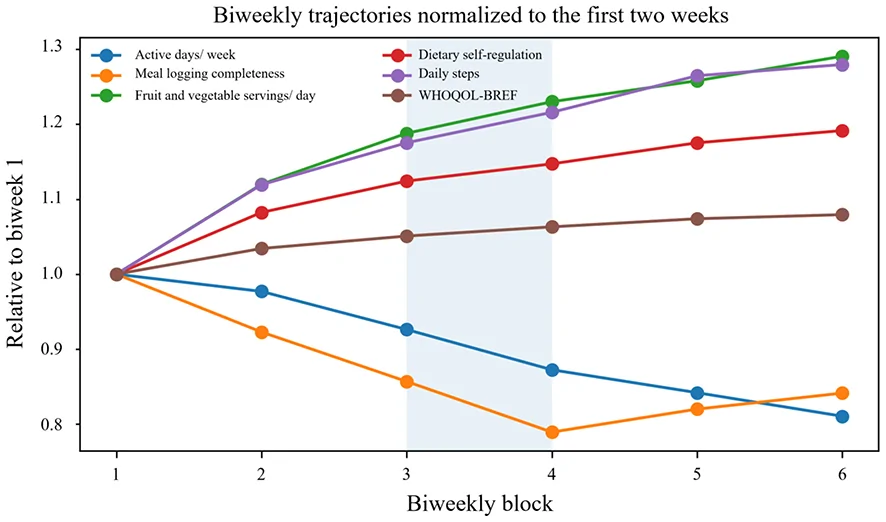
Normalized biweekly Phase 1 implementation, behavioral, activity, and quality-of-life trajectories.

Figure 4 shows initial improvement followed by partial stabilization in the normalized biweekly trajectories. The pattern is descriptive and does not identify an unmeasured mechanism or a specific mid-semester cause.

The biweekly aggregation used to summarize longitudinal change, as depicted in Table 4, helps minimize week-to-week variability.

**Table 4:** Biweekly phase 1 summary. Values are means with 95% confidence intervals after participant-level aggregation within each two-week block.

Biweek	Weeks	Active days	Meal logging	Fruit/veg	Self-regulation	Steps	WHOQOL
1	1–2	5.97 (5.90, 6.04)	0.552 (0.539, 0.566)	3.33 (3.20, 3.46)	66.43 (64.33, 68.53)	6,503 (6,163, 6,843)	71.40 (69.50, 73.30)
2	3–4	5.83 (5.76, 5.90)	0.510 (0.498, 0.521)	3.73 (3.59, 3.86)	71.90 (69.77, 74.03)	7,279 (6,938, 7,620)	73.85 (71.93, 75.76)
3	5–6	5.53 (5.45, 5.60)	0.473 (0.460, 0.487)	3.95 (3.81, 4.10)	74.68 (72.48, 76.87)	7,643 (7,300, 7,987)	75.03 (73.12, 76.94)
4	7–8	5.21 (5.12, 5.29)	0.436 (0.423, 0.448)	4.09 (3.95, 4.24)	76.20 (74.01, 78.38)	7,908 (7,554, 8,262)	75.91 (73.98, 77.84)
5	9–10	5.03 (4.95, 5.10)	0.453 (0.440, 0.466)	4.19 (4.03, 4.34)	78.06 (75.88, 80.25)	8,225 (7,860, 8,590)	76.68 (74.75, 78.60)
6	11–12	4.84 (4.77, 4.91)	0.465 (0.452, 0.477)	4.30 (4.13, 4.46)	79.15 (76.91, 81.39)	8,322 (7,957, 8,687)	77.07 (75.08, 79.06)

As illustrated in Table 5, the weekly mixed-effects models quantify the longitudinal pattern of Phase 1 outcomes and the mid-semester attenuation in implementation measures. These estimates describe change over time in a single-arm cohort and do not establish that the intervention, calendar time, or any individual component caused the observed changes.

**Table 5:** Random-intercept linear mixed-effects models for weekly Phase 1 outcomes. The week is coded as 0 for week 1; mid-semester indicates weeks 6–8.

Outcome	Week effect per study week (95% CI)	Mid-semester effect (95% CI)
Active days/week	−0.120 (−0.129, − 0.112); *p* < 0.001	−0.059 (−0.125, 0.006); *p* 0.076
Meal logging completeness	−0.009 (−0.010, −0.007); *p* < 0.001	−0.046 (−0.057, −0.035); *p* < 0.001
Fruit and vegetable servings/day	0.090 (0.086, 0.094); *p* < 0.001	0.113 (0.080, 0.146); *p* < 0.001
Dietary self-regulation	1.189 (1.134, 1.243); *p* < 0.001	1.147 (0.713, 1.582); *p* < 0.001
Daily steps	173.0 (163.3, 182.7); *p* < 0.001	144.1 (66.9, 221.3); *p* < 0.001
WHOQOL-BREF	0.532 (0.491, 0.574); *p* < 0.001	0.588 (0.255, 0.920); *p* < 0.001

Paired analyses from baseline to week 12 confirmed the endpoint pattern. Within-participant improvements were observed in fruit and vegetable consumption, dietary self-regulation, daily steps, and WHOQOL-BREF scores, while BMI remained statistically and clinically stable. Wilcoxon sensitivity analyses yielded the same substantive interpretation for the behavioral and quality-of-life endpoints. These paired estimates are not interpreted as causal treatment effects.

As shown in Table 6, improvements from baseline to week 12 were observed in behavioral and quality-of-life endpoints, with stable BMI.

**Table 6:** Baseline-to-week-12 paired outcome analyses.

Outcome	Baseline mean ±SD	Week 12 mean ±SD	Mean change (95% CI)	Paired t *p*	Wilcoxon *p*	Cohen dz
Fruit and vegetable intake (servings/day)	3.20 ± 0.90	4.30 ± 1.15	+1.10 (+1.00, +1.20)	< 0.001	< 0.001	1.47
Dietary self-regulation (0–100)	64.58 ± 15.18	79.45 ± 16.07	+14.87 (+13.65, +16.09)	< 0.001	< 0.001	1.70
Daily steps	6,278 ± 2,480	8,338 ± 2,574	+2,060 (+1,844, +2,275)	< 0.001	< 0.001	1.33
WHOQOL-BREF overall (0–100)	70.74 ± 13.47	77.21 ± 14.20	+6.47 (+5.65, +7.28)	< 0.001	< 0.001	1.11
BMI (kg/m^2^)	22.41 ± 3.09	22.40 ± 3.07	−0.01 (−0.06, +0.04)	0.788	0.810	−0.02

**Figure 5:**
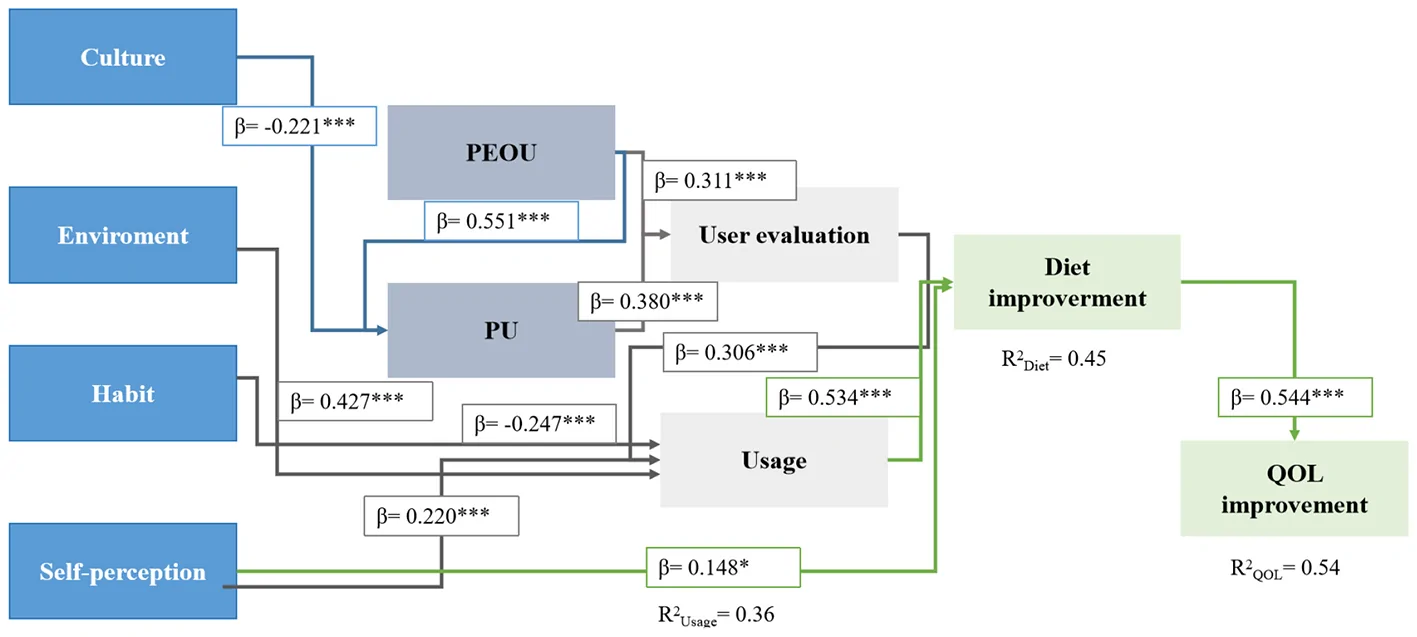
CEHSV-KANO-TAM-SEM contextual pathway model.

The Phase 1 contextual adoption model showed good fit (chi-square/df = 1.87, CFI = 0.98, RMSEA = 0.032) and explained 36% of the variance in usage, 45% in dietary improvement, and 54% in quality-of-life improvement (Table 7 and Figure 5). These SEM estimates are associative, not causal mediation effects. Externalized health culture was associated with lower perceived usefulness (beta = −0.221, *p* < 0.001), living environment with higher usage (beta = 0.427, *p* < 0.001), and habit intensity with lower usage (beta = −0.247, *p* < 0.001) and less dietary improvement (beta = −0.193, *p* < 0.01). Self-perception/self-efficacy was positively associated with usage and dietary improvement. Within the TAM chain, PEOU was associated with PU (beta = 0.551, *p* < 0.001), followed by associations from PU to user evaluation, user evaluation to usage, usage to dietary improvement, and dietary improvement to quality-of-life improvement.

**Table 7:** CEHSV-KANO-TAM-SEM model fit, standardized path estimates, and subgroup contrasts.

Component/path	Estimate	Interpretation
Global fit	chi-square/df = 1.87; CFI = 0.98; RMSEA = 0.032	Strong overall fit
R2 usage	0.36	Contextual and TAM constructs explained 36% of variance in usage
R2 dietary improvement	0.45	Usage and related model paths explained 45% of variance in dietary improvement
R2 QOL improvement	0.54	Dietary improvement and related model paths explained 54% of variance in quality-of-life improvement
Culture → PU	beta = −0.221, *p* < 0.001	Externalized health culture was associated with lower perceived usefulness
Environment → Usage	beta = 0.427, *p* < 0.001	Living environment was associated with greater usage
Habit → Usage	beta = −0.247, *p* < 0.001	Greater habit burden was associated with lower sustained use
Habit → Diet improvement	beta = −0.193, *p* < 0.01	Greater habit burden was associated with less dietary improvement
Self-efficacy → Usage	beta = 0.220, *p* < 0.001	Higher self-efficacy was associated with greater usage
Self-efficacy → Diet improvement	beta = 0.148, *p* < 0.05	Higher self-efficacy was associated with greater dietary improvement
PEOU → PU	beta = 0.551, *p* < 0.001	Ease of use was associated with higher perceived usefulness
PU → User evaluation	beta = 0.380, *p* < 0.001	Perceived usefulness was associated with more favorable user evaluation
User evaluation → Usage	beta = 0.306, *p* < 0.001	More favorable user evaluation was associated with greater usage
Usage → Diet improvement	beta = 0.534, *p* < 0.001	Higher usage was associated with greater dietary improvement
Diet improvement → QOL improvement	beta = 0.544, *p* < 0.001	Greater dietary improvement was associated with greater quality-of-life improvement.

As the subgroup analyses were exploratory, based on a small sample, and involved multiple path comparisons without multiplicity adjustment, they should be interpreted as hypothesis-generating. While measurement invariance across living environments was supported at the tested levels, suggesting no instability in the measurement and structural specifications across dormitory- and home-living groups, this does not establish equivalence. In the self-efficacy-stratified models, the environment-to-usage coefficient was numerically higher in the group with low self-efficacy (β = 0.51 vs. 0.41; *p* for difference = 0.07), whereas the habit-to-usage (β = −0.31 vs. −0.23; *p* for difference = 0.04) and user-evaluation-to-usage (β = 0.24 vs. 0.40; *p* for difference = 0.04) coefficients differed only slightly. The usage-to-dietary-change contrast was not detected (β = 0.55 vs. 0.52; *p* for difference = 0.61). These estimates do not demonstrate effect modification and may reflect sampling variation or multiplicity. However, they do identify candidate contextual and self-efficacy contingencies for preregistered testing in a sufficiently powered study.

**Figure 6:**
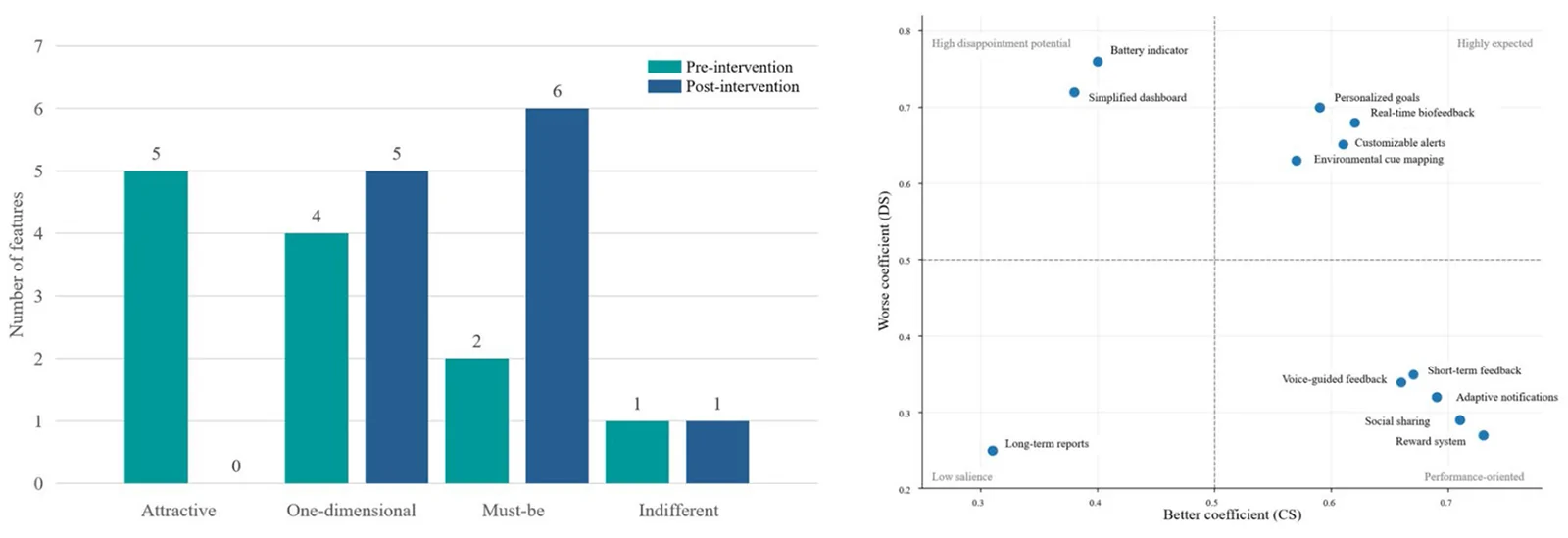
Baseline-to-week-12 Kano feature reclassification. Feature-level Kano coefficients show which functions were classified as expected infrastructure vs. performance features at the two assessment points.

As shown in Figure 6 and Table 8, Kano feature classifications differed between baseline and week 12. At baseline, the 12-feature portfolio comprised five attractive features, four one-dimensional features, two must-be features, and one indifferent feature. At week 12, the distribution was 0 attractive features, 5 one-dimensional features, 6 must-be features, and 1 indifferent feature. Real-time biofeedback, personalized goal notifications, customizable alerts, and environmental cue mapping were reclassified as must-be features; short-term outcome feedback, social sharing, adaptive notification frequency, reward systems, and voice-guided feedback were reclassified as one-dimensional features; and long-term progress reports remained indifferent. Because Kano data were collected at only two time points, the study demonstrates only baseline-to-week-12 reclassification; it cannot determine the speed, monotonicity, durability, or causal mechanism of the change in expectations.

**Table 8:** Baseline-to-week-12 Kano feature reclassification over the 12-week intervention.

Feature	Baseline category	CS	DS	Week 12 category	Trajectory
Real-time biofeedback visualization	One-dimensional	0.62	0.68	Must-be	O → M
Personalized goal notifications	One-dimensional	0.59	0.70	Must-be	O → M
Short-term outcome feedback	Attractive	0.67	0.35	One-dimensional	A → O
Social sharing	Attractive	0.71	0.29	One-dimensional	A → O
Adaptive notification frequency	Attractive	0.69	0.32	One-dimensional	A → O
Simplified data dashboard	Must-be	0.38	0.72	Must-be	M → M
Reward system	Attractive	0.73	0.27	One-dimensional	A → O
Customizable alerts	One-dimensional	0.61	0.65	Must-be	O → M
Long-term progress reports	Indifferent	0.31	0.25	Indifferent	I → I
Voice-guided feedback	Attractive	0.66	0.34	One-dimensional	A → O
Environmental cue mapping	One-dimensional	0.57	0.63	Must-be	O → M
Battery life indicator	Must-be	0.40	0.76	Must-be	M → M

### 3.3 In Phase 2, AbSound showed feasible wear and alert acknowledgment, with exploratory directional behavioral estimates.

Change-score estimates were examined for all six participant-level outcomes—late-night snacking, eating speed, diet quality, SREB, weight, and waist circumference; these were exploratory pilot estimates, not efficacy estimates.

The Phase 2 randomized pilot included 16 participants per group, all retained in the analysis. Baseline values were broadly similar across groups, but the pilot was not powered to establish baseline equivalence. AbSound participants wore the device for a mean of 9.35 hours/day (SD 1.98) across 25.81 days (SD 2.21) of the 28-day pilot. These metrics support short-term daily-use feasibility under open-label conditions. Participant-level AbSound exposure, alert interaction, and apparent algorithmic agreement estimates are reported in Table 9.

**Table 9:** AbSound pilot device feasibility and participant-level algorithmic performance.

Device metric	Mean ±SD	95% CI	*n*
Daily wear time (h/day)	9.35 ± 1.98	8.30 to 10.40	16
Days worn out of 28	25.81 ±2.21	24.63 to 26.99	16
Hunger-timing alerts	22.06 ±7.22	18.22 to 25.91	16
Fullness/pace alerts	18.19 ± 5.09	15.48 to 20.90	16
Alerts acknowledged (%)	78.54 ± 6.70	74.97 to 82.12	16
Eating episodes detected	75.44 ± 6.87	71.78 to 79.10	16
Participant-level algorithmic accuracy (%)	88.77 ± 2.77	87.29 to 90.25	16

**Figure 7:**
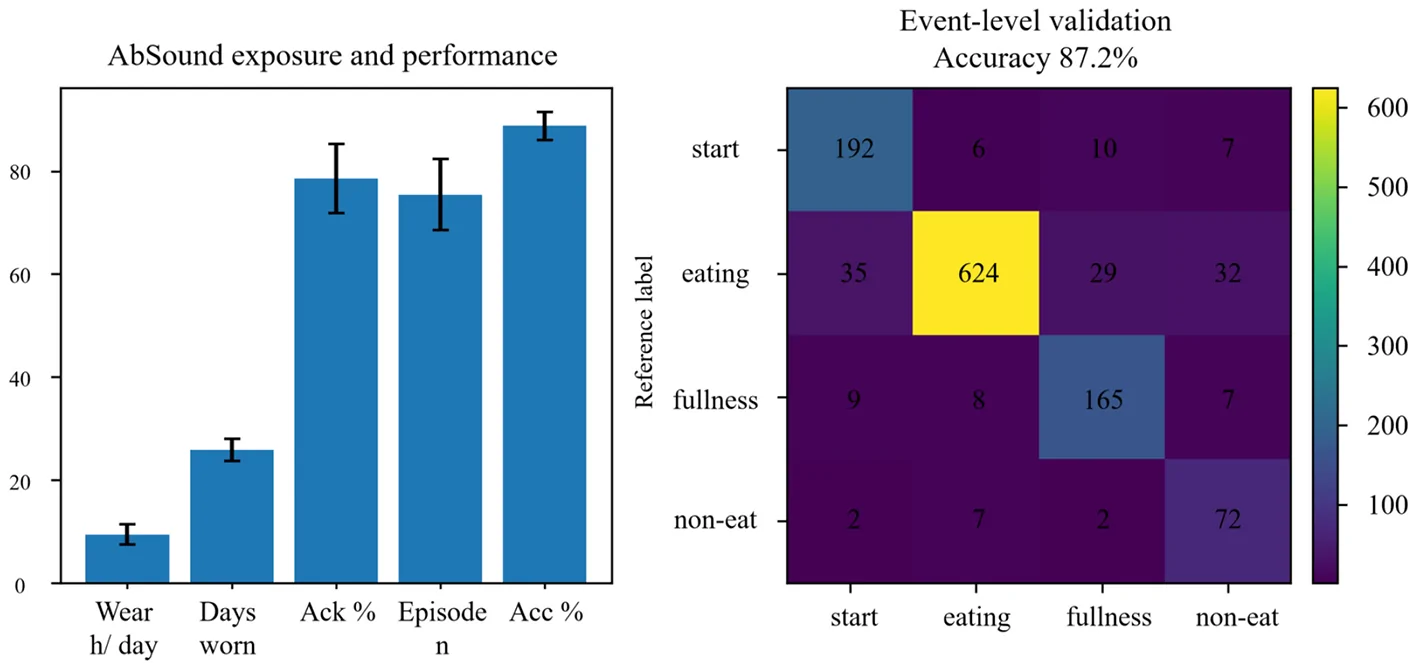
AbSound exposure, participant-level performance indicators, and event-level confusion matrix.

Figure 7 summarizes wear and alert-interaction feasibility and descriptive event-classification agreement. It does not provide participant-disjoint external validation or construct validation of hunger, satiety, or gut motility.

Table 10 shows class-specific variation in performance. Because events were participant-clustered and the reference labels were not independent physiological ground truth, the metrics are descriptive agreement estimates.

**Table 10:** Event-level AbSound classification metrics from 1,207 labeled acoustic events. Because events are clustered within participants, these values are descriptive validation metrics.

Reference class	*n*	Precision	Recall/sensitivity	Specificity	F1
meal_start	215	0.807	0.893	0.954	0.848
eating_episode	720	0.967	0.867	0.957	0.914
post_meal_fullness	189	0.801	0.873	0.960	0.835
non_eating_gut_sound	83	0.610	0.867	0.959	0.716
Overall	1,207		87.2% accuracy		Macro-F1 82.8%

**Figure 8:**
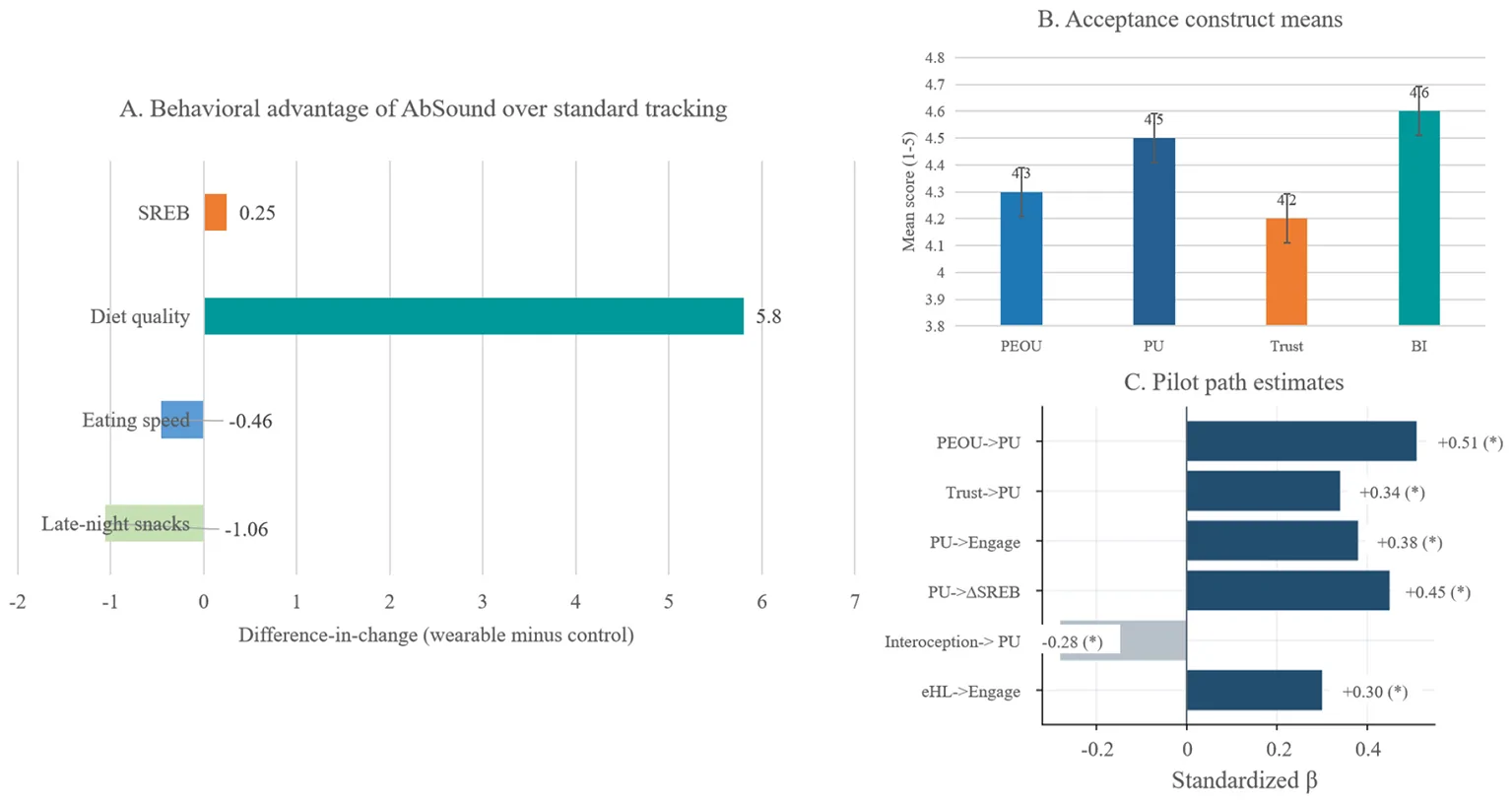
Phase 2 directional between-group change estimates, pilot acceptance construct means, and exploratory pilot path estimates.

Table 11 presents exploratory between-group change-score estimates for short-term decision-process outcomes. The sensitivity power analysis showed that 16 participants per group provided 80% power only for effects of approximately *d* = 1.02. Given the small sample, four-week follow-up, open-label design, multiple outcomes, and no multiplicity adjustment, p values and Hedges g should be interpreted as directional estimates for trial planning rather than confirmatory evidence of efficacy. Figure 8 presents the exploratory Phase 2 between-group difference-in-change estimates for the four behavioral outcomes; the anthropometric outcomes are reported in Table 11.

**Table 11:** Phase 2 randomized feasibility pilot outcomes. Between-group estimates are exploratory and directional; p values and Hedges g are reported for estimation and trial planning, not efficacy testing.

Outcome	AbSound baseline	AbSound follow-up	AbSound change	Control baseline	Control follow-up	Control change	Difference in change (95% CI)	*p*	Hedges g
Late-night snacks/week	3.29 ± 0.92	2.07 ± 1.05	−1.22	2.98 ±1.01	2.82 ± 1.39	−0.16	−1.06 (−1.51, −0.61)	< 0.001	−1.68
Eating speed (1 slow-5 fast)	3.48 ± 0.36	2.97 ± 0.37	−0.51	3.27 ±0.62	3.22 ± 0.69	−0.05	−0.46 (−0.67, −0.25)	< 0.001	−1.53
Diet quality (0–100)	59.60 ± 8.64	67.80 ± 12.15	+8.20	61.70 ± 5.73	64.10 ± 6.06	+2.40	+5.80 (+2.72, +8.88)	< 0.001	1.33
SREB	2.94 ± 0.59	3.35 ± 0.56	+0.41	2.83 ± 0.29	2.99 ± 0.35	+0.16	+0.25 (+0.04, +0.46)	0.023	0.83
Weight (kg)	60.20 ± 5.21	60.10 ± 5.22	−0.10	59.50 ± 8.10	60.00 ± 7.87	+0.50	−0.60 (−1.18, −0.02)	0.043	−0.73
Waist circumference (cm)	80.30 ± 5.58	79.90 ± 5.49	−0.40	78.90 ± 9.13	79.30 ± 9.15	+0.40	−0.80 (−1.65, +0.04)	0.062	−0.67

Across phases, the data support feasibility and longitudinal decision-process associations. Phase 1 provides within-participant evidence, and Phase 2 provides randomized feasibility estimates with exploratory directional signals. The combined findings do not establish intervention efficacy.

## 4 Discussion

The 12-week field intervention was associated with within-participant improvements in dietary self-regulation, fruit and vegetable intake, daily steps, and quality of life, while BMI remained stable. The weekly models indicated a progression, coinciding with declines in engagement and meal logging, particularly during the mid-semester period. Given that Phase 1 was single-arm, these trajectories constitute longitudinal process evidence and, as such, cannot isolate an intervention effect.

Large within-participant effects may be indicative of repeated assessment, self-monitoring, Hawthorne and novelty effects, the timing of academic semesters, regression to the mean, or selective follow-up. Phase 1 evaluated the multicomponent platform, whereas abdominal acoustic feedback was evaluated only in Phase 2; therefore, it would be erroneous to attribute the trajectories from Phase 1, or the contextual SEM, specifically to AbSound, or to interpret them as causal mediation.

Phase 2 wear time, days worn, and alert acknowledgment supported short-term feasibility. Change-score estimates directionally favored AbSound for late-night snacking, eating speed, diet quality, and SREB. However, with 16 participants per group, four-week follow-up, an open-label design, multiple outcomes, and power limited to very large effects, the estimates are imprecise and potentially unstable; they should inform the design of a definitive trial rather than support efficacy claims.

Event-level accuracy (87.2%), balanced accuracy (87.5%), and macro-F1 (82.8%) quantify agreement with composite meal-state/acoustic-event labels. Because events were clustered within participants and no independent physiological reference standard was used, these metrics do not establish construct validity for hunger, satiety, or gut motility. Table 12 therefore positions AbSound's contribution as a field-tested, closed-loop behavioral prompting workflow rather than superior physiological sensing.

**Table 12:** Comparison of AbSound with selected abdominal acoustic sensing and bowel-sound analysis systems.

System/study	Sensing and deployment	Analytic or validation focus	Interaction role and evidentiary boundary
Wang et al. (45)	Dual MEMS sensor heads; quiet fasting and post-meal recordings	CNN bowel-sound detection; manually labeled data and an across-participant test; pre/post-meal acoustic features	Acoustic monitoring and feature analysis; no closed-loop dietary prompting or self-regulation intervention.
Ding et al. (46)	Wearable four-PMUT prototype with real-time LabVIEW display	Bench characterization of sensitivity, noise resolution, and non-linearity	Sensor characterization and waveform display; no behavioral event classification or dietary intervention.
Kutsumi et al. (47)	Smartphone built-in microphone; recordings from 100 participants	CNN/LSTM bowel-sound recognition and a bowel-sound interval proxy for motility	Portable bowel-sound recognition and motility-proxy analysis; no meal-contingent behavioral feedback.
Mansour et al. (48)	Lightweight multichannel SonicGuard; phantom tests and recordings from 10 patients	Hardware qualification against digital stethoscopes and exploratory bowel-activity classification	Clinical/research abdominal-sound recording; no real-time behavioral feedback or eating-behavior intervention.
Baronetto et al. (49)	Smart T-shirt with embedded microphones; 136 h from 27 participants	Efficient-U-Net event spotting in highly imbalanced continuous audio; leave-one-participant-out validation	Wearable event detection; evidence is limited to signal-processing performance.
Yu et al. (50)	Labeled and unlabeled bowel-sound datasets; laboratory model development	Branchformer recognition with self-supervised pretraining under limited labeled data	Acoustic recognition model only; no user-interaction or behavioral-feedback component.
AbSound (present study)	Epigastric wearable plus companion app; four-week randomized feasibility pilot	Agreement with composite meal-state/acoustic-event labels (1,207 non-independent events); no physiological reference standard	Closed-loop behavioral support using meal-timing acoustic cues; feasibility-level evidence only; not a diagnostic or validated gut-motility system.

The knowledge-behavior and gut-brain interpretations remain hypothesis-generating because knowledge, appetite hormones, microbiome composition, neurocognitive processes, and gastric motility were neither measured nor manipulated. The outcomes therefore concern behavior and self-regulation, not confirmation of a biological mechanism.

The HCI findings suggest three testable design priorities: low-friction, comprehensible prompts; adaptive burden reduction when logging declines; and reliable infrastructure as initially attractive features become expected. Two-point Kano reclassification cannot establish a universal or durable trajectory, and the prompt data do not identify which interface component produced behavioral change. Component-level micro-randomized evaluation is required.

Construct validity of alert timing remains unestablished because no validated, time-locked subjective appetite ratings were available. The study-defined labels indicate meal-related temporal contexts, not experienced hunger or satiety. Misclassification can occur in both directions: non-eating gut sounds may trigger mistimed prompts, whereas meal-relevant events may be missed. False-positive prompts may reduce perceived relevance and trust or increase notification fatigue; false negatives may reduce perceived usefulness. Future trials should pair acoustic event windows with repeated validated appetite ratings, use participant-disjoint external validation, and quantify the user-experience consequences of false and missed alerts before stronger mechanistic or clinical claims are made.

These findings should also be situated within evidence linking diet, physical activity, and sleep with mental health in young adults and linking dietary quality with mental health and academic outcomes in university students (61–63). Mobile dietary assessment, clinical artificial intelligence, and precision public-health research similarly emphasize transparent measurement validity, human oversight, and appropriately bounded inference (64–67). Future definitive trials and reports should follow relevant AI, pilot-trial, and digital-health reporting guidance (54–57, 68).

### 4.1 Limitations

The study has six principal limitations. First, Phase 1 lacked a control group and is vulnerable to maturation, semester effects, self-monitoring, regression to the mean, and selective follow-up. Second, 48.5% meal-logging completeness creates adherence and differential-exposure bias, but exposure was not linked to all outcome records with sufficient granularity for adherence-adjusted analysis. Third, dietary outcomes were self-reported. Fourth, Phase 2 was small, open-label, four weeks long, and tested multiple outcomes; it estimates feasibility and unstable directional signals rather than efficacy. Fifth, subgroup path contrasts were exploratory, underpowered, and not adjusted for multiplicity. Sixth, acoustic performance was evaluated against composite participant-clustered labels rather than participant-disjoint external data, validated appetite ratings, or physiological reference standards. Misclassification of non-eating gut sounds may therefore reduce alert precision and user trust. Generalizability is limited to digitally literate Taiwanese university students and requires replication across age, cultural, clinical, and digital-literacy groups.

## 5 Conclusion

The 12-week context-aware digital nutrition intervention was associated with within-participant improvements in dietary self-regulation, fruit and vegetable intake, activity, and quality of life, but its single-arm design precludes causal attribution. The separate four-week randomized pilot established short-term wear and alert-interaction feasibility and yielded exploratory directional signals for late-night snacking, eating speed, diet quality, and SREB relative to standard tracking. AbSound should therefore be interpreted as a behavioral support system rather than a validated appetite or physiological monitor. A larger, adequately powered randomized trial should incorporate time-locked validated appetite measures, participant-disjoint acoustic validation, and longer-term outcomes.

## Data Availability

The aggregated data supporting the conclusions of this article are included in the article. Deidentified participant-level behavioral, wearable-sensor, and acoustic-event data are not publicly available because due to the privacy of the participants and the conditions of ethics approval. Requests for access may be directed to Tungjing Fang (ftj@ntut.edu.tw) and will be considered for qualified researchers subject to institutional review and an appropriate data-use agreement.
